# Differences in quality of life between genders in acromegaly

**DOI:** 10.1002/edm2.229

**Published:** 2021-02-03

**Authors:** Daniel Ballesteros‐Herrera, Paulina Briseño‐Hernández, Rodrigo Pérez‐Esparza, Lesly A. Portocarrero‐Ortiz

**Affiliations:** ^1^ Department of Neurosurgery Instituto Nacional de Neurología y Neurocirugía, MVS Mexico City Mexico; ^2^ Universidad Nacional Autónoma de México Mexico City Mexico; ^3^ Department of Neuropsychiatry INNN Mexico City Mexico; ^4^ Department of Neuroendocrinology INNN Mexico City Mexico

**Keywords:** acromegaly, anxiety, depression, gender, quality of life

## Abstract

**Objective:**

To study the impact of secondary mental disorders in patients affected with acromegaly and correlate them with quality of life (QoL) and disease status.

**Design:**

An observational transversal descriptive and comparative study that evaluates QoL's impact due to secondary mental disorders in affected Mexicans with acromegaly using AcroQoL and SF‐36 instruments. Correlation of the results with the disease's biochemical status was performed. According to Beck's scales, anxiety and depression analyses evaluate QoL's impact, and its gender variability is described.

**Results:**

Eighty‐five patients with diagnoses of acromegaly were included. The mean age was 43.18 years, 47 being women (55.29%). The mean age at diagnosis was 37.95 years, with no difference between men and women. AcroQoL and SF‐36 global and sub‐domain scores differed significantly between men and women, the latter having lower global and individual sub‐domain scores. The mean score of QoL, according to AcroQoL, is 59.40. In women, the mean values are less (55.13) than men (64.68), *p* = 0.021. The sub‐domain analyses’ scores in physical, appearance and social relationships were less in women (53.21; 47.34; 62.32) than men (62.68; 56.76; 73.87) *p* = 0.044, 0.069 and 0.013, respectively. Higher Beck's Depression Inventory (BDI) and Beck's Anxiety Inventory (BAI) scores correlated with lower QoL as assessed by global and individual sub‐domain scores. Women presented significantly higher BDI and BAI mean scores when compared to men regardless of their biochemical status. Anxiety (*p* = 0.027) and depression (*p* < 0.001) severity were higher in women compared to men.

**Conclusion:**

Correlations between female gender, depression/anxiety scores and QoL require further validation. There is much to be routinely done to improve secondary psychopathology in patients affected by this disease. The need for mental status screening at diagnosis should be emphasized to identify secondary mental illnesses to improve QoL with its treatment.

## Graphical abstract

Quality of life is something that no one cares to measure within illnesses. We found a profound affection in patients with acromegaly. We measured these secondary psychiatric disorders with standardized psychiatric scales to evidence them and their correlation with the status of the disease

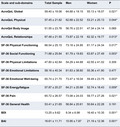

## 1 INTRODUCTION

Acromegaly is a rare disease caused by the hypersecretion of growth hormone (GH) and a secondary insulin‐like factor 1 (IGF‐1) secretion, in most cases, due to a secreting presence of pituitary adenoma.^1^ Prevalence has been estimated, ranging between 2.8 and 13.7 cases per 100,000 population, affecting males and females almost equally.^2^ The Mexican Acromegaly Registry (MAR) has registered more than 2,000 cases of the disease, estimating a regional prevalence of 18 cases per million population. A greater prevalence in women compared to men, commonly associated with glucose abnormalities and hypertension.^3^

Health‐related quality of life (QoL) has been used as an outcome in clinical practice that assesses patients’ perception of their well‐being and functioning. Although numerous QoL measurement instruments have been developed, the Acromegaly Quality of Life Questionnaire (AcroQoL) addresses some specific items regarding disease progression and treatment outcomes.^4^ Several factors have been identified as impacting QoL in acromegaly, including depression, body mass index (BMI),^5^ body image, general psychopathology, pain, comorbidities and type of treatment.^6^, ^7^ Biochemical markers of treatment outcomes have not been consistently associated with a change in QoL scores. Overall, the results of most studies regarding predictors of QoL in acromegaly are ambiguous or insufficient.^5^

This study aimed to describe the QoL in treated patients with acromegaly in the Mexican National Institute of Neurology and Neurosurgery.

## 2 MATERIAL AND METHODS

An observational transversal descriptive and comparative study that included patients with acromegaly. The evaluation of QoL with AcroQoL, SF‐36 and secondary psychopathology with BDI and BAI scales was performed with the patient's previous consent. Results were correlated with gender, disease's biochemical status, comorbidities, tumoral size and type of treatment.

### 2.1 Patients

Patients were older than 18 years with a diagnosis of acromegaly based on the Second Mexican Consensus (age‐ and sex‐adjusted IGF‐1 levels in combination with GH nadir during an oral glucose tolerance test with cut‐offs of >0.5 ng/dL^7^), who were attending follow‐up in the Neuroendocrinology clinic of the INNN from 2011 to 2014. All patients were included regardless of age at diagnosis.

Patients were divided into two groups: biochemically active and controlled (defined as a glucose‐suppressed GH below <0.5 ng/mL and a normal IGF‐1).^4^, ^8^

Quality of life was assessed using the AcroQoL and the 36‐Item Short Form Survey (SF‐36). Additionally, Beck's Depression Inventory (BDI)^9^ and Beck's Anxiety Inventory (BAI) were filled out by the patients to evaluate these psychiatric symptom domains.

AcroQol consists of 22 items, each punctuated with a maximum of 5 points. Results are reported from 22 (worst QoL) to 110 (best QoL). To interpret the results in a range of 0 −100, standardization with a mathematical adjustment was performed (Y = ([X‐min] x 100) /max‐min)).

SF‐36 comprises 36 items that evaluate 8 dimensions: physical functioning, physical role, pain, general health, vitality, social functioning, emotional and mental health. The range for each dimension is evaluated from 0 to 100. Depending on the number of categories, each answer is worth a different percentage to complete a maximum of 100 (example=3 categories=0–50–100). The average of each category gives the result of each dimension. The average of all dimensions gives the final result. The closest to 100 is the best, and 0 is the worst.

BDI consists of 21 items, each punctuated with a maximum of 3 points, from 0 (best) to 63 points (worst). According to results, patients are classified in minimum (0–9), mild (10–16), moderate (17–29), and severe (30–36). As same as BDI, BAI is punctuated from 0 to 3 for every 21 items, classifying patients, as stated earlier.

Sociodemographic and disease‐related variables were acquired using the medical records.

Results were presented as means (SDs). Normality was examined using the Shapiro–Wilk test, and Spearman's test assessed the association of clinical variables with AcroQol, SF‐36, BDI and BAI scores.

The present study was approved by the Ethics and Scientific Committees of the National Institute of Neurology and Neurosurgery (INNN) in Mexico City. Written informed consent of all subjects was obtained before participation.

## 3 RESULTS

Eighty‐five patients with a diagnosis of acromegaly were included. The mean age was 43.18 years ±10.79, 47 being women (55.29%). The mean age at diagnosis was 37.95 ± 13.59 years, with no difference between men and women.

### 3.1 QoL and sociodemographic characteristics

**FIGURE 1:**
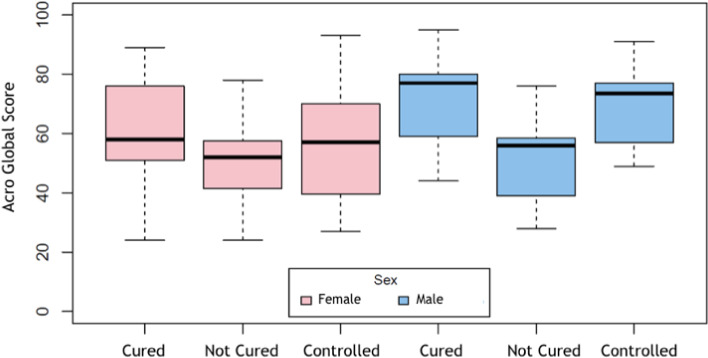
Correlation between AcroQol Global Score and patient's disease status. A significant lower total score in females compared to males in any status

AcroQoL and SF‐36 global and sub‐domain scores (AcroQoL Physical, AcroQoL Relationships; SF‐36 Physical functioning, SF‐36 Social functioning, SF‐36 Emotional well‐being, SF‐36 Energy/fatigue and SF‐36 Pain) differed significantly between men and women (Figure 1), the latter having lower global and individual sub‐domain scores. Mean QoL value, according to AcroQoL, is 59.40. In women, the mean values are less (55.13) than men (64.68) *p* = 0.021. In the sub‐domain analyses, physical, appearance and social relationships mean scores in women were less (53.21; 47.34; 62.32), compared to men (62.68; 56.76; 73.87) *p* = 0.044, 0.069 and 0.013, respectively. Women were also more commonly unemployed (57.45%; *p* = 0.002), correlating with lower physical AcroQoL scores (*p* = 0.043). Age, marital status and years of education did not differ between sex and were not associated with QoL scores.

### 3.2 QoL and clinical characteristics

At diagnosis, age was 44.51 ± 10.91 years for women and 41.53 ± 10.55 years for men, with no difference between genders – age was not associated with different QoL scores. Tumour size was available for 80 patients. 17 were microadenomas and 63 macroadenomas. There was no relation between tumour size and QoL scores.

### 3.3 QoL and biochemical markers

Considering GH and IGF‐1 cut‐off values to define cured, active, or discordant acromegaly, only cured acromegaly was associated with global AcroQoL scores (*p* = 0.05). Cured patients presented a better score in vitality and pain items of SF‐36 compared with active patients (*p* = 0.060, and *p* = 0.057) with no statistical significance.

### 3.4 QoL and mental health

When comparing BDI (16.40 ± 10.35 vs. 9.34 ± 6.98, *p* = 0.001) and BAI (21.19 ± 12.36 vs. 10.95 ± 7.87) scores between women and men, higher scores correlated with lower QoL as assessed by global, and all sub‐domain scores in AcroQoL (*p*=<0.001) regardless of their biochemical status (Table 1). When assessing severity, 40.43% of women presented moderate to severe depression, compared to 15.79% in men (*p* < 0.001). Moderate to severe anxiety was present in 68.83% of women, compared to 21.05% in men (*p* = 0.027).

**TABLE 1:** Quality of life, depression and anxiety scores in men and women

Scale and sub‐domains	Total Sample	Men	Women	*p*
AcroQoL Global	59.40 ± 19.06	64.68 ± 19.15	55.13 ± 18.07	0.021*
AcroQoL Physical	57.45 ± 21.62	62.68 ± 22.52	53.21 ± 20.13	0.044*
AcroQol Body Image	51.55 ± 23.76	56.76 ± 22.51	47.34 ± 24.14	0.069
AcroQoL Relationships	67.48 ± 21.55	73.87 ± 22.19	62.32 ± 19.77	0.013*
SF−36 Physical Functioning	66.54 ± 25.15	73.18 ± 24.85	61.17 ± 24.34	0.017*
SF−36 Social Functioning	71.88 ± 25.84	81.79 ± 19.83	63.87 ± 27.49	0.003*
SF−36 Physical Limitations	47.80 ± 42.84	54.29 ± 44.68	42.55 ± 41.02	0.309
SF−36 Emotional Limitations	58.16 ± 40.34	61.63 ± 38.60	55.36 ± 41.90	0.477
SF−36 Emotional Well‐being	65.74 ± 21.70	73.47 ± 16.04	59.49 ± 23.75	0.003*
SF−36 Energy/fatigue	57.87 ± 20.27	64.21 ± 20.88	52.74 ± 18.43	0.009*
SF−36 Pain	65.72 ± 26.57	73.08 ± 24.03	59.77 ± 27.26	0.021*
SF−36 General Health	53.41 ± 21.65	56.84 ± 20.61	50.64 ± 22.28	0.191
BDI	13.25 ± 9.62	9.34 ± 6.98	16.40 ± 10.35	0.001*
BAI	16.61 ± 11.71	10.95 ± 7.87	21.19 ± 12.36	0.001*

Abbreviations: 36‐Item Short Form Survey; Beck's Depression Inventory; AcroQol, Acromegaly Quality of Life Questionnaire; BAI, Beck's Anxiety Inventory.

* Significant ‘*p*’ values.

### 3.5 QoL and comorbidity

Comorbidity information was available for 81 patients of the total sample. Diabetes, glucose abnormalities, hypothyroidism, hypogonadism, hypopituitarism, hypocortisolism, hypertension and dyslipidaemia were considered important associated comorbidities. Of the 81 patients, 31 were comorbidity free; 11 presented 1; 14 presented 2; and 25 presented >3. There was no correlation between the number of comorbid diseases and QoL. However, when assessed individually, the presence of diabetes was associated with lower scores in the body image and relationships sub‐domains of the AcroQoL (*p* = 0.027; *p* = 0.003, respectively).

### 3.6 QoL and type of treatment

Information for treatment type and duration was only available for 80 patients. 68.75% were treated with surgery using the transsphenoidal approach (microscopic and endoscopic endonasal). No correlation was found between modality of treatment (radiosurgery, surgical approach, radiotherapy or a combination of the previous) and QoL.

## 4 DISCUSSION

Acromegaly is a disease that deteriorates QoL and diminishes life expectancy because of the accompanying comorbidities. Patients can suffer cognitive deficits, emotional changes, social impairment, high levels of frustration, uncertainty, low functional and social adaptability.

As stated by the WHO, QoL is the individual perception of self‐existence in the cultural context. The system of values where existence is based, it's constituted on people's objectives, expectancies and social norms. QoL is influenced by health, psychologic and social status.^10^ Based on this definition, we consider QoL as a phenomenon affected by both the disease itself and its treatment because of the potential secondary adverse effects that can be presented during follow‐up.

AcroQoL (Webb) and a posterior study in 2005 confirmed in English native speakers the severe impairment of QOL, making it a must evaluation during acromegaly approach.^10^, ^11^ Nienke et al. in 2004 showed that radiotherapy worsened QoL importantly despite its healing effect confirmed by SF‐36 and the Nottingham Health Profile questionnaires.^12^ Nonetheless, in our results, we found no significant difference in this matter (*p* = 0.83). Another study by Wolters found in a prospective design after 2.5 years of follow‐up a significant decline in QoL in acromegaly patients. Moreover, in 2017 a systematic review by Geraedts studied the specific predictors of QoL in acromegaly with the currently available data in the literature. 51 of 1162 studies were included detailing the heterogeneity of their designs. Highlighting the need for longitudinal studies where QoL is long termed assessed throughout the different phases of the disease and that evaluate the effect of psychologic therapies on the affected. In our study using some well‐known and validated psychiatric questionnaires to assess QoL,^13^, ^14^, ^15^ we found that BMI and higher depression scores harmed QoL,^16^, ^17^, ^18^ being consistent with theirs, but no consensus was met on other predictors such as age, gender and biochemical status. We found a marked prevalence of lower scores in women, related to higher anxiety and depression scores, and markedly lower AcroQoL global scores with statistically significant differences in appearance and physical effects consistent with the previously reported English literature.^10^

Matta et al. in 2008^11^ evaluated QoL using SF‐36 and found that the most affected item was physical appearance. In our results, appearance was the most affected with a score of 47.34 ± 24.14 in women and 62.88 ± 22.52 in men despite biochemical status. The less affected was personal relationships with a score of 62.32 ± 19.77, following the presented study. SF‐36 results displayed differences between cured and non‐cured patients, being the latter the ones with lower scores. We found no statistically significant differences in physical performance, and general health status, like Van der Klaw study.^19^ One of the latest studies studying the effects of acromegaly in QoL by Solomon et al.^20^ reported executive function compromise in an observational case–control study.

Psychologic morbidity is present in a significant percentage of patients with acromegaly, especially women. This association found between QoL and female gender has been reported in other previous studies, with no difference between treatment modalities.^21^, ^22^, ^23^ Nonetheless, no consensus has been reached. In Mexico, Garduño^24^ highlighted that women had worse global scores with no correlation between GH levels, tumoral size, disease status and QoL. Our study found lower scores in all sub‐domains in females compared to males with a statistically significant difference. Our least affected item was interpersonal relationships.

Female patients suffer depression and lower self‐esteem due to the morphologic changes derived from the disease.^25^ Another aspect that seems to contribute is fatigue that can affect daily activities and work performance. During our correlation of depressive symptoms with the QoL questionnaires,^26^ negative coefficients reflected a greater number of depressive symptoms indicative of QoL deterioration. The detriment of QoL is not only explained by biochemical disease status but also by secondary psychopathology. Our findings are similar to Geraedts, reaffirming that evaluation of depressive and anxious symptoms, which are modifiable, would improve the patients’ management, and therefore QoL.^5^, ^6^, ^21^, ^26^ Women appear to have a higher severity of psychiatric disorders than men. Probably because of their rigorous self‐assessment and self‐approbation. We also consider that Mexican idiosyncrasy could influence these results. Nonetheless, this has not been studied extensively. No previous reports have assessed anxiety levels as our study, an important matter to our psychologic assessment consideration.

## 5 STUDY LIMITATIONS

The principal study limitation is its transversal nature. We also had one evaluation after treatment, where the only thing we could compare is QoL as a group and between cured and non‐cured during the evaluation. We consider our sample as representative of our population. Nonetheless, it is convenient to perform these questionnaires to every patient diagnosed with acromegaly from the first evaluation and during follow‐up. Longitudinal studies during the course of the disease are needed to solve these matters and clarify what we know about secondary psychopathology.

## 6 CONCLUSION

Correlations between female gender, depression/anxiety scores and QoL require further validation with multi‐centric studies. Secondary psychopathology should be included during the initial assessment of patients with acromegaly. It should emphasize the need for mental status screening at acromegaly diagnosis, especially in women, because of the high incidence and severity of secondary mental illnesses in this particular population. Confirming the high negative impact, it confers to QoL.

## CONFLICT OF INTEREST

This research did not receive any specific grant from any funding agency in the public, commercial or not‐for‐profit sector. No conflict of interest could be perceived as prejudicing the impartiality of the research reported.

## AUTHOR CONTRIBUTIONS

Lesly A. Portocarrero‐Ortiz contributed to idea conception, supervision in manuscript elaboration and participated in the discussion. Daniel Ballesteros‐Herrera contributed to redaction of the manuscript, translation to English, participated in the discussion and publication logistics. Paulina Briseño‐Hernández contributed to questionnarie application. Rodrigo Pérez‐Esparza contributed to statistical analysis and participated in the discussion.

## Data Availability

The data that support the findings of this study are available on request from the corresponding author. The data are not publicly available due to privacy and ethical restrictions under the Mexican law that protects personal data.
